# 4-Cyclo­propyl-1-(6′-de­oxy-1′,2′-*O*-iso­propyl­idene-α-d-gluco­furanos­yl)-1*H*-1,2,3-triazole

**DOI:** 10.1107/S1600536813021351

**Published:** 2013-08-07

**Authors:** Qiurong Zhang, Peng He, Guangqiang Zhou, Kang Yu, Hongmin Liu

**Affiliations:** a New Drug Reseach & Development Center, Zhengzhou Univresity, Zhengzhou 450001, People’s Republic of China

## Abstract

In the title compound, C_14_H_21_N_3_O_5_, the tetra­hydro­furan ring adopts an envelope conformation with the C atom bearing the substituent as the flap. The penta­furan­ose ring adopts a twisted conformation about the C—C bond fusing the rings. The dihedral angle between these rings (all atoms), which are *cis* fused, is 72.89 (14)°. The cyclo­propane ring is disordered over two orientations in a 0.576 (5):0.424 (5) ratio; the dihedral angles subtended to the triazole ring are 53.3 (11) and 46.6 (9)°, respectively. In the crystal, the mol­ecules are linked by O—H⋯N and O—H⋯O hydrogen bonds, generating (001) sheets. A weak C—H⋯O inter­action also occurs.

## Related literature

For further synthetic details, see: Pradere *et al.* (2008 ▶). For background to 1,2,3-triazoles, see: Alvarez *et al.* (1994 ▶); Genin *et al.* (2000 ▶).
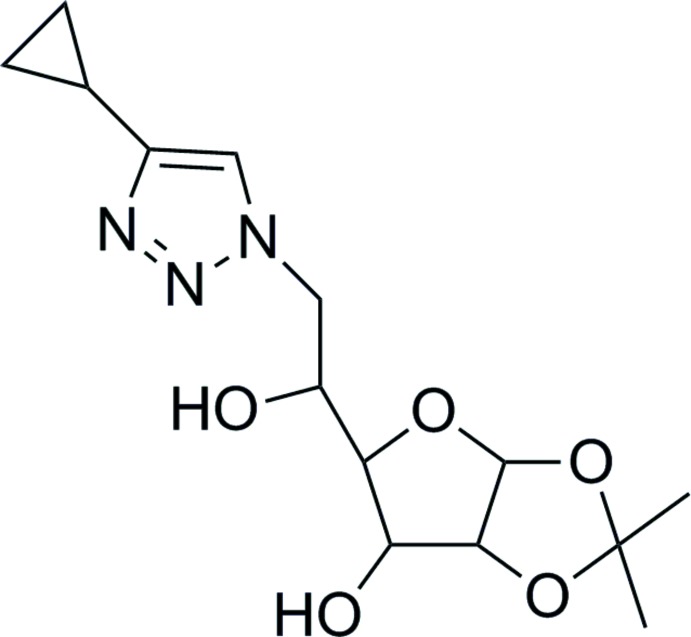


## Experimental

### 

#### Crystal data

C_14_H_21_N_3_O_5_
*M* *_r_* = 311.34
Orthorhombic, 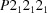

*a* = 8.5905 (3) Å
*b* = 8.7215 (3) Å
*c* = 20.7373 (7) Å
*V* = 1553.68 (9) Å^3^
*Z* = 4
Cu *K*α radiation
μ = 0.85 mm^−1^
*T* = 291 K
0.22 × 0.2 × 0.18 mm

#### Data collection

Bruker MWPC diffractometer
Absorption correction: multi-scan (*SADABS*; Bruker, 2004 ▶) *T*_min_ = 0.835, *T*_max_ = 0.862
5692 measured reflections
2778 independent reflections
2503 reflections with *I* > 2σ(*I*)
*R*_int_ = 0.028

#### Refinement

*R*[*F* ^2^ > 2σ(*F* ^2^)] = 0.042
*wR*(*F* ^2^) = 0.113
*S* = 1.06
2778 reflections
220 parameters
2 restraints
H atoms treated by a mixture of independent and constrained refinement
Δρ_max_ = 0.25 e Å^−3^
Δρ_min_ = −0.20 e Å^−3^

### 

Data collection: *FRAMBO* (Bruker, 2004 ▶); cell refinement: *SAINT* (Bruker, 2004 ▶); data reduction: *SAINT*; program(s) used to solve structure: *SHELXS97* (Sheldrick, 2008 ▶); program(s) used to refine structure: *SHELXL97* (Sheldrick, 2008 ▶); molecular graphics: *OLEX2* (Dolomanov *et al.*, 2009 ▶); software used to prepare material for publication: *OLEX2*.

## Supplementary Material

Crystal structure: contains datablock(s) I, global. DOI: 10.1107/S1600536813021351/hb7111sup1.cif

Structure factors: contains datablock(s) I. DOI: 10.1107/S1600536813021351/hb7111Isup2.hkl

Additional supplementary materials:  crystallographic information; 3D view; checkCIF report

## Figures and Tables

**Table 1:** Hydrogen-bond geometry (Å, °)

*D*—H⋯*A*	*D*—H	H⋯*A*	*D*⋯*A*	*D*—H⋯*A*
O3—H3⋯N3^i^	0.83 (2)	1.95 (2)	2.767 (3)	171 (3)
O5—H5⋯O3^ii^	0.82 (3)	2.02 (3)	2.821 (3)	164 (3)
C7—H7⋯O1^iii^	0.93	2.59	3.496 (3)	165

Symmetry codes: (i) 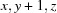; (ii) 
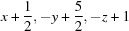
; (iii) 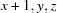.

**Fig. 1.**
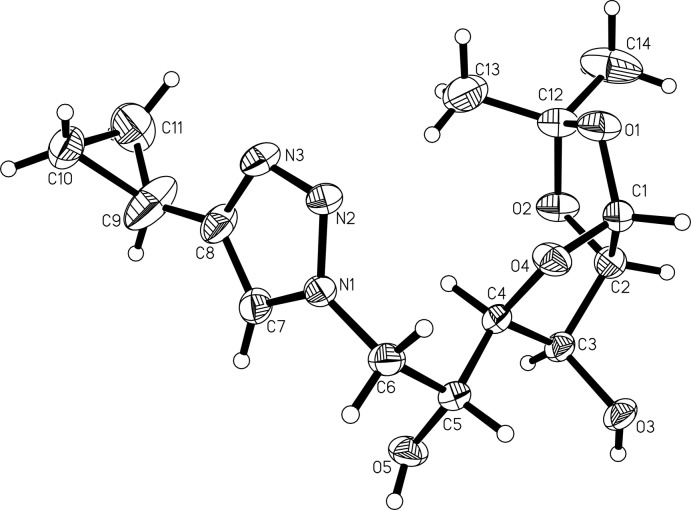
The molecular structure of (I) showing 30% probability displacement ellipsoids.

**Fig. 2.**
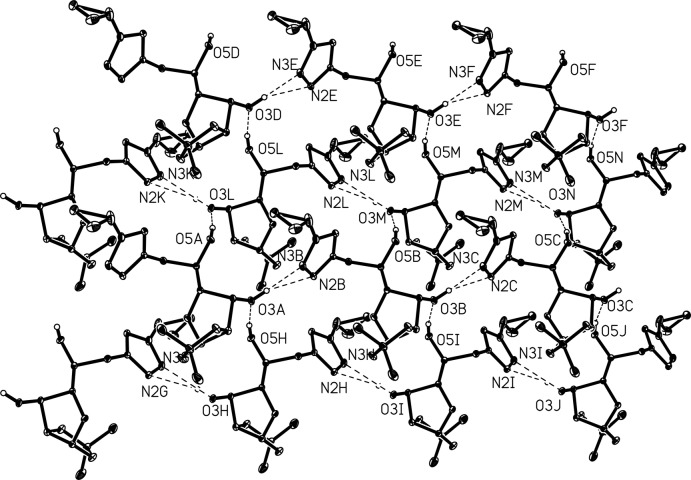
Packing diagram for (I).

## supplementary crystallographic information

### 1. Comment

1,2,3-Triazoles have been shown to have various biological activities, such as anti-HIV (Alvarez *et al.*, 1994) and antibacterial (Genin *et al.*, 2000). C_14_H_21_N3_O_5, the title compound (I), is a new 1,2,3-triazole. The nucleus of the molecule consists of one methylenedioxy rings, one 1,2,3-triazole ring, one cyclopropyl ring and one tetrahydrofuran ring (Fig. 1). The tetrahydrofuran ring fuses with one methylenedioxy ring,having the *cis* arrangement at the ring junctions and giving a V-shaped molecule.

The crystal packing, which features O—H···N hydrogen bonds (Table 1), is shown in Figure 2.

### 2. Experimental

The title compound (I) was synthesized from 6-azido-6-deoxy-1,2-*O*-isopropylidene-alpha -*D*-glucofuranose, whose starting material was D-glucose. The copper catalyzed reaction of 6-azido-6-deoxy-1,2-*O*-isopropylidene-alpha -*D*-glucofuranose(1 mmol) and cyclopropylacetylene (1.2 mmol) in water/tetrahydrofuran (2 ml:2 ml) was stirred for 3 h at room temperature. The mixture was filtered and evaporated. and the residue extracted with EtOAc (50 ml). The organic layer was washed brine, dried over Na_2_SO_4_ for 6 h, filtered, and the solvent evaporated *in vacuo*. Purification of the residue by column chromatography gave the title compound as white solid.

Colourless prisms were grown by slow evaporation from acetone solution at room temperature for two weeks. mp:389–391k; *R*_f_ = 0.30 (petroleum ether/EtOAc, 1:1); ^1^H NMR (400 MHz, DMSO-d_6_)σ: 7.69(1*H*, s), 5.86(1*H*, d, *J* = 3.6 Hz), 5.27(1*H*, d, *J* = 4.9 Hz), 5.19(1*H*, d, *J* = 6.4 Hz), 4.47(1*H*, dt, *J* = 10.2, 5.1 Hz), 4.42(1*H*, d, *J* = 3.6 Hz), 4.23(1*H*, d, *J* = 14.0, 8.0 Hz), 4.08–3.93(2*H*, m), 3.75(1*H*, dd, *J* = 8.8, 2.5 Hz), 1.92(1*H*, dq, *J* = 8.4, 5.0 Hz), 1.37(3*H*, s), 1.24(3*H*, s), 0.94–0.82(2*H*, m), 0.79–0.63(2*H*, m); ^13^C NMR (100 MHz, DMSO-d_6_) σ: 153.61, 126.82, 115.95, 109.77, 89.90, 86.23, 78.05, 71.49, 58.80, 31.88, 31.39, 12.70, 11.72.

### 3. Refinement

All H atoms were placed geometrically and treated as riding on their parent atoms with C—H are 0.96 Å (methylene) or 0.93 Å (aromatic), 0.82 Å (hydroxyl)and *U*_iso_(H) =1.2*U*_eq_(C).

Attempts to confirm the absolute structure by refinement of the Flack parameter in the presence of 1156 sets of Friedel equivalents led to an inconclusive value of 0.0 (3). Therefore, the absolute configuration was assigned to correspond with that of the known chiral centres in a precursor molecule, which remained unchanged during the synthesis of the title compound.

### Crystal data

**Table:**

C_14_H_21_N_3_O_5_	*D*_x_ = 1.331 Mg m^−^^3^
*M**_r_* = 311.34	Melting point = 389–391 K
Orthorhombic, *P*2_1_2_1_2_1_	Cu *K*α radiation, λ = 1.54178 Å
*a* = 8.5905 (3) Å	Cell parameters from 2697 reflections
*b* = 8.7215 (3) Å	θ = 4.3–67.0°
*c* = 20.7373 (7) Å	µ = 0.85 mm^−^^1^
*V* = 1553.68 (9) Å^3^	*T* = 291 K
*Z* = 4	PRISMATIC, colourless
*F*(000) = 664	0.22 × 0.2 × 0.18 mm

### Data collection

**Table:**

Bruker MWPC diffractometer	2778 independent reflections
Radiation source: fine-focus sealed tube	2503 reflections with *I* > 2σ(*I*)
Graphite monochromator	*R*_int_ = 0.028
Detector resolution: 0 pixels mm^-1^	θ_max_ = 67.1°, θ_min_ = 4.3°
phi and ω scans	*h* = −10→6
Absorption correction: multi-scan (*SADABS*; Bruker, 2004)	*k* = −6→10
*T*_min_ = 0.835, *T*_max_ = 0.862	*l* = −24→22
5692 measured reflections	

### Refinement

**Table:**

Refinement on *F*^2^	Secondary atom site location: difference Fourier map
Least-squares matrix: full	Hydrogen site location: inferred from neighbouring sites
*R*[*F*^2^ > 2σ(*F*^2^)] = 0.042	H atoms treated by a mixture of independent and constrained refinement
*wR*(*F*^2^) = 0.113	*w* = 1/[σ^2^(*F*_o_^2^) + (0.0567*P*)^2^ + 0.1063*P*] where *P* = (*F*_o_^2^ + 2*F*_c_^2^)/3
*S* = 1.06	(Δ/σ)_max_ < 0.001
2778 reflections	Δρ_max_ = 0.25 e Å^−^^3^
220 parameters	Δρ_min_ = −0.20 e Å^−^^3^
2 restraints	Extinction correction: *SHELXL*, Fc^*^=kFc[1+0.001xFc^2^λ^3^/sin(2θ)]^-1/4^
Primary atom site location: structure-invariant direct methods	Extinction coefficient: 0.0020 (3)

### Special details

**Table:**

Geometry. All e.s.d.'s (except the e.s.d. in the dihedral angle between two l.s. planes) are estimated using the full covariance matrix. The cell e.s.d.'s are taken into account individually in the estimation of e.s.d.'s in distances, angles and torsion angles; correlations between e.s.d.'s in cell parameters are only used when they are defined by crystal symmetry. An approximate (isotropic) treatment of cell e.s.d.'s is used for estimating e.s.d.'s involving l.s. planes.
Refinement. Refinement of *F*^2^ against ALL reflections. The weighted *R*-factor *wR* and goodness of fit *S* are based on *F*^2^, conventional *R*-factors *R* are based on *F*, with *F* set to zero for negative *F*^2^. The threshold expression of *F*^2^ > σ(*F*^2^) is used only for calculating *R*-factors(gt) *etc*. and is not relevant to the choice of reflections for refinement. *R*-factors based on *F*^2^ are statistically about twice as large as those based on *F*, and *R*- factors based on ALL data will be even larger.

### Fractional atomic coordinates and isotropic or equivalent isotropic displacement parameters (Å^2^)

**Table:**

	*x*	*y*	*z*	*U*_iso_*/*U*_eq_	Occ. (<1)
O1	−0.0805 (2)	0.9329 (2)	0.39252 (9)	0.0556 (5)	
O2	0.0625 (2)	1.0684 (2)	0.31968 (8)	0.0541 (5)	
O3	0.2108 (2)	1.32136 (18)	0.44356 (9)	0.0487 (4)	
O4	0.12445 (19)	0.99779 (18)	0.46166 (8)	0.0470 (4)	
O5	0.5249 (2)	1.1144 (2)	0.44763 (9)	0.0517 (4)	
N1	0.4448 (2)	0.7779 (2)	0.46218 (9)	0.0395 (4)	
N2	0.3396 (2)	0.6662 (2)	0.45796 (11)	0.0461 (5)	
N3	0.3860 (3)	0.5760 (2)	0.41084 (11)	0.0536 (5)	
C1	−0.0064 (3)	1.0493 (3)	0.42728 (12)	0.0427 (5)	
H1	−0.0800	1.1009	0.4561	0.051*	
C2	0.0544 (3)	1.1602 (3)	0.37614 (12)	0.0458 (5)	
H2	−0.0119	1.2508	0.3708	0.055*	
C3	0.2187 (3)	1.1997 (3)	0.39848 (11)	0.0398 (5)	
H3A	0.2871	1.2245	0.3621	0.048*	
C4	0.2639 (2)	1.0486 (2)	0.43038 (11)	0.0378 (5)	
H4	0.2922	0.9745	0.3968	0.045*	
C5	0.3927 (2)	1.0555 (2)	0.47975 (11)	0.0401 (5)	
H5A	0.3625	1.1273	0.5139	0.048*	
C6	0.4246 (3)	0.8990 (3)	0.51006 (11)	0.0427 (5)	
H6A	0.5179	0.9056	0.5363	0.051*	
H6B	0.3387	0.8723	0.5382	0.051*	
C7	0.5573 (3)	0.7605 (3)	0.41813 (13)	0.0486 (6)	
H7	0.6429	0.8238	0.4115	0.058*	
C8	0.5199 (4)	0.6308 (3)	0.38513 (14)	0.0560 (7)	
C9	0.6279 (7)	0.5539 (8)	0.3376 (4)	0.0575 (13)	0.576 (5)
H9	0.7207	0.6118	0.3247	0.069*	0.576 (5)
C10	0.5544 (9)	0.4602 (11)	0.2880 (4)	0.0937 (19)	0.576 (5)
H10A	0.4419	0.4512	0.2890	0.112*	0.576 (5)
H10B	0.5990	0.4632	0.2450	0.112*	0.576 (5)
C11	0.6422 (10)	0.3805 (10)	0.3380 (4)	0.076 (2)	0.576 (5)
H11A	0.7418	0.3361	0.3262	0.091*	0.576 (5)
H11B	0.5842	0.3241	0.3703	0.091*	0.576 (5)
C12	−0.0353 (3)	0.9372 (3)	0.32677 (12)	0.0537 (6)	
C13	0.0573 (5)	0.7939 (4)	0.31160 (18)	0.0822 (10)	
H13A	0.1480	0.7901	0.3386	0.123*	
H13B	0.0886	0.7957	0.2672	0.123*	
H13C	−0.0060	0.7051	0.3194	0.123*	
C14	−0.1795 (4)	0.9543 (5)	0.28580 (19)	0.0976 (14)	
H14A	−0.2461	0.8672	0.2922	0.146*	
H14B	−0.1503	0.9606	0.2412	0.146*	
H14C	−0.2338	1.0460	0.2980	0.146*	
C9A	0.5637 (10)	0.5566 (12)	0.3225 (5)	0.0575 (13)	0.424 (5)
H9A	0.5328	0.6124	0.2835	0.069*	0.424 (5)
C10A	0.7242 (11)	0.4953 (13)	0.3224 (5)	0.0937 (19)	0.424 (5)
H10C	0.7798	0.4917	0.3631	0.112*	0.424 (5)
H10D	0.7883	0.5136	0.2847	0.112*	0.424 (5)
C11A	0.5835 (14)	0.3960 (15)	0.3149 (7)	0.076 (2)	0.424 (5)
H11C	0.5634	0.3524	0.2727	0.091*	0.424 (5)
H11D	0.5550	0.3307	0.3509	0.091*	0.424 (5)
H5	0.591 (3)	1.141 (4)	0.4739 (12)	0.059 (9)*	
H3	0.266 (3)	1.393 (3)	0.4302 (14)	0.058 (8)*	

### Atomic displacement parameters (Å^2^)

**Table:**

	*U*^11^	*U*^22^	*U*^33^	*U*^12^	*U*^13^	*U*^23^
O1	0.0553 (10)	0.0571 (10)	0.0543 (9)	−0.0242 (9)	0.0018 (8)	0.0009 (8)
O2	0.0620 (11)	0.0563 (10)	0.0439 (8)	−0.0223 (9)	−0.0041 (8)	0.0044 (8)
O3	0.0526 (9)	0.0281 (7)	0.0655 (11)	−0.0076 (7)	0.0121 (8)	−0.0079 (7)
O4	0.0394 (8)	0.0445 (8)	0.0573 (9)	−0.0097 (7)	−0.0010 (7)	0.0133 (7)
O5	0.0420 (9)	0.0476 (9)	0.0654 (11)	−0.0149 (7)	−0.0044 (8)	0.0029 (8)
N1	0.0358 (9)	0.0308 (8)	0.0521 (10)	−0.0021 (7)	−0.0022 (8)	0.0047 (8)
N2	0.0431 (10)	0.0332 (9)	0.0621 (11)	−0.0064 (8)	0.0011 (9)	0.0018 (9)
N3	0.0657 (13)	0.0322 (9)	0.0628 (12)	−0.0073 (10)	0.0019 (10)	−0.0037 (9)
C1	0.0357 (10)	0.0382 (11)	0.0542 (12)	−0.0002 (9)	0.0036 (10)	−0.0022 (10)
C2	0.0435 (12)	0.0362 (11)	0.0579 (14)	0.0003 (10)	−0.0021 (10)	0.0052 (10)
C3	0.0414 (11)	0.0291 (10)	0.0488 (12)	−0.0028 (9)	0.0066 (10)	−0.0005 (9)
C4	0.0369 (10)	0.0283 (10)	0.0483 (11)	−0.0003 (8)	0.0021 (9)	−0.0038 (9)
C5	0.0384 (11)	0.0320 (10)	0.0498 (11)	−0.0040 (9)	−0.0006 (9)	−0.0061 (9)
C6	0.0415 (12)	0.0397 (11)	0.0470 (11)	−0.0034 (9)	−0.0059 (9)	0.0011 (10)
C7	0.0426 (12)	0.0357 (11)	0.0676 (15)	−0.0003 (10)	0.0094 (11)	0.0072 (10)
C8	0.0673 (17)	0.0349 (11)	0.0658 (15)	0.0006 (11)	0.0148 (13)	0.0022 (11)
C9	0.049 (4)	0.0495 (16)	0.074 (4)	0.001 (3)	0.006 (3)	−0.003 (2)
C10	0.074 (3)	0.127 (5)	0.080 (3)	0.012 (4)	0.000 (3)	−0.046 (4)
C11	0.089 (6)	0.047 (2)	0.091 (6)	0.017 (4)	0.022 (4)	−0.001 (4)
C12	0.0585 (15)	0.0525 (14)	0.0502 (12)	−0.0192 (12)	−0.0062 (12)	0.0014 (12)
C13	0.091 (2)	0.0624 (19)	0.093 (2)	−0.0184 (18)	0.019 (2)	−0.0190 (18)
C14	0.092 (3)	0.107 (3)	0.093 (2)	−0.047 (2)	−0.042 (2)	0.038 (2)
C9A	0.049 (4)	0.0495 (16)	0.074 (4)	0.001 (3)	0.006 (3)	−0.003 (2)
C10A	0.074 (3)	0.127 (5)	0.080 (3)	0.012 (4)	0.000 (3)	−0.046 (4)
C11A	0.089 (6)	0.047 (2)	0.091 (6)	0.017 (4)	0.022 (4)	−0.001 (4)

### Geometric parameters (Å, º)

**Table:**

O1—C1	1.399 (3)	C7—C8	1.361 (4)
O1—C12	1.418 (3)	C8—C9	1.511 (8)
O2—C2	1.420 (3)	C8—C9A	1.499 (12)
O2—C12	1.428 (3)	C9—H9	0.9800
O3—C3	1.416 (3)	C9—C10	1.459 (10)
O3—H3	0.829 (18)	C9—C11	1.517 (11)
O4—C1	1.405 (3)	C10—H10A	0.9700
O4—C4	1.432 (3)	C10—H10B	0.9700
O5—C5	1.413 (3)	C10—C11	1.458 (11)
O5—H5	0.821 (18)	C11—H11A	0.9700
N1—N2	1.332 (3)	C11—H11B	0.9700
N1—C6	1.460 (3)	C12—C13	1.514 (4)
N1—C7	1.338 (3)	C12—C14	1.509 (4)
N2—N3	1.316 (3)	C13—H13A	0.9600
N3—C8	1.355 (4)	C13—H13B	0.9600
C1—H1	0.9800	C13—H13C	0.9600
C1—C2	1.527 (3)	C14—H14A	0.9600
C2—H2	0.9800	C14—H14B	0.9600
C2—C3	1.525 (3)	C14—H14C	0.9600
C3—H3A	0.9800	C9A—H9A	0.9800
C3—C4	1.525 (3)	C9A—C10A	1.479 (12)
C4—H4	0.9800	C9A—C11A	1.420 (17)
C4—C5	1.509 (3)	C10A—H10C	0.9700
C5—H5A	0.9800	C10A—H10D	0.9700
C5—C6	1.528 (3)	C10A—C11A	1.495 (18)
C6—H6A	0.9700	C11A—H11C	0.9700
C6—H6B	0.9700	C11A—H11D	0.9700
C7—H7	0.9300		
			
C1—O1—C12	110.60 (18)	C8—C9—H9	116.7
C2—O2—C12	109.78 (18)	C8—C9—C11	119.2 (6)
C3—O3—H3	108 (2)	C10—C9—C8	116.3 (5)
C1—O4—C4	109.89 (16)	C10—C9—H9	116.7
C5—O5—H5	110 (2)	C10—C9—C11	58.6 (5)
N2—N1—C6	119.59 (19)	C11—C9—H9	116.7
N2—N1—C7	111.23 (19)	C9—C10—H10A	117.5
C7—N1—C6	129.17 (19)	C9—C10—H10B	117.5
N3—N2—N1	106.30 (19)	H10A—C10—H10B	114.6
N2—N3—C8	109.8 (2)	C11—C10—C9	62.7 (5)
O1—C1—O4	113.18 (19)	C11—C10—H10A	117.5
O1—C1—H1	110.7	C11—C10—H10B	117.5
O1—C1—C2	104.92 (19)	C9—C11—H11A	117.9
O4—C1—H1	110.7	C9—C11—H11B	117.9
O4—C1—C2	106.35 (17)	C10—C11—C9	58.7 (5)
C2—C1—H1	110.7	C10—C11—H11A	117.9
O2—C2—C1	103.44 (18)	C10—C11—H11B	117.9
O2—C2—H2	112.9	H11A—C11—H11B	115.1
O2—C2—C3	109.4 (2)	O1—C12—O2	106.32 (19)
C1—C2—H2	112.9	O1—C12—C13	108.8 (3)
C3—C2—C1	104.40 (18)	O1—C12—C14	108.6 (3)
C3—C2—H2	112.9	O2—C12—C13	109.3 (2)
O3—C3—C2	109.04 (18)	O2—C12—C14	110.2 (3)
O3—C3—H3A	111.8	C14—C12—C13	113.3 (3)
O3—C3—C4	111.92 (19)	C12—C13—H13A	109.5
C2—C3—H3A	111.8	C12—C13—H13B	109.5
C2—C3—C4	99.90 (17)	C12—C13—H13C	109.5
C4—C3—H3A	111.8	H13A—C13—H13B	109.5
O4—C4—C3	104.53 (17)	H13A—C13—H13C	109.5
O4—C4—H4	109.0	H13B—C13—H13C	109.5
O4—C4—C5	108.58 (18)	C12—C14—H14A	109.5
C3—C4—H4	109.0	C12—C14—H14B	109.5
C5—C4—C3	116.50 (18)	C12—C14—H14C	109.5
C5—C4—H4	109.0	H14A—C14—H14B	109.5
O5—C5—C4	106.52 (18)	H14A—C14—H14C	109.5
O5—C5—H5A	108.7	H14B—C14—H14C	109.5
O5—C5—C6	111.98 (18)	C8—C9A—H9A	115.6
C4—C5—H5A	108.7	C10A—C9A—C8	113.0 (7)
C4—C5—C6	112.02 (17)	C10A—C9A—H9A	115.6
C6—C5—H5A	108.7	C11A—C9A—C8	123.5 (10)
N1—C6—C5	112.82 (18)	C11A—C9A—H9A	115.6
N1—C6—H6A	109.0	C11A—C9A—C10A	62.1 (8)
N1—C6—H6B	109.0	C9A—C10A—H10C	118.1
C5—C6—H6A	109.0	C9A—C10A—H10D	118.1
C5—C6—H6B	109.0	C9A—C10A—C11A	57.0 (7)
H6A—C6—H6B	107.8	H10C—C10A—H10D	115.3
N1—C7—H7	127.3	C11A—C10A—H10C	118.1
N1—C7—C8	105.5 (2)	C11A—C10A—H10D	118.1
C8—C7—H7	127.3	C9A—C11A—C10A	60.9 (7)
N3—C8—C7	107.2 (2)	C9A—C11A—H11C	117.7
N3—C8—C9	128.4 (3)	C9A—C11A—H11D	117.7
N3—C8—C9A	113.7 (4)	C10A—C11A—H11C	117.7
C7—C8—C9	123.5 (4)	C10A—C11A—H11D	117.7
C7—C8—C9A	137.3 (4)	H11C—C11A—H11D	114.8
C9A—C8—C9	24.4 (3)		
			
O1—C1—C2—O2	−21.9 (2)	C2—O2—C12—O1	−11.0 (3)
O1—C1—C2—C3	−136.45 (19)	C2—O2—C12—C13	−128.3 (3)
O2—C2—C3—O3	164.93 (18)	C2—O2—C12—C14	106.5 (3)
O2—C2—C3—C4	−77.6 (2)	C2—C3—C4—O4	−38.2 (2)
O3—C3—C4—O4	77.1 (2)	C2—C3—C4—C5	−158.00 (19)
O3—C3—C4—C5	−42.7 (3)	C3—C4—C5—O5	−59.1 (2)
O4—C1—C2—O2	98.2 (2)	C3—C4—C5—C6	178.16 (19)
O4—C1—C2—C3	−16.3 (2)	C4—O4—C1—O1	106.0 (2)
O4—C4—C5—O5	−176.68 (17)	C4—O4—C1—C2	−8.7 (2)
O4—C4—C5—C6	60.6 (2)	C4—C5—C6—N1	50.0 (2)
O5—C5—C6—N1	−69.6 (2)	C6—N1—N2—N3	179.2 (2)
N1—N2—N3—C8	−0.2 (3)	C6—N1—C7—C8	−179.0 (2)
N1—C7—C8—N3	0.1 (3)	C7—N1—N2—N3	0.3 (3)
N1—C7—C8—C9	−170.2 (4)	C7—N1—C6—C5	65.6 (3)
N1—C7—C8—C9A	163.2 (6)	C7—C8—C9—C10	−155.2 (6)
N2—N1—C6—C5	−113.0 (2)	C7—C8—C9—C11	137.6 (6)
N2—N1—C7—C8	−0.2 (3)	C7—C8—C9A—C10A	70.7 (11)
N2—N3—C8—C7	0.1 (3)	C7—C8—C9A—C11A	141.4 (9)
N2—N3—C8—C9	169.7 (4)	C8—C9—C10—C11	−109.7 (7)
N2—N3—C8—C9A	−167.5 (5)	C8—C9—C11—C10	104.7 (7)
N3—C8—C9—C10	36.6 (9)	C8—C9A—C10A—C11A	116.9 (11)
N3—C8—C9—C11	−30.5 (8)	C8—C9A—C11A—C10A	−100.3 (9)
N3—C8—C9A—C10A	−127.0 (8)	C9—C8—C9A—C10A	5.6 (10)
N3—C8—C9A—C11A	−56.2 (10)	C9—C8—C9A—C11A	76.4 (16)
C1—O1—C12—O2	−4.1 (3)	C12—O1—C1—O4	−99.3 (2)
C1—O1—C12—C13	113.5 (2)	C12—O1—C1—C2	16.3 (3)
C1—O1—C12—C14	−122.7 (3)	C12—O2—C2—C1	20.2 (3)
C1—O4—C4—C3	30.3 (2)	C12—O2—C2—C3	131.0 (2)
C1—O4—C4—C5	155.31 (18)	C9A—C8—C9—C10	−22.7 (12)
C1—C2—C3—O3	−84.9 (2)	C9A—C8—C9—C11	−89.8 (16)
C1—C2—C3—C4	32.6 (2)		

### Hydrogen-bond geometry (Å, º)

**Table:**

*D*—H···*A*	*D*—H	H···*A*	*D*···*A*	*D*—H···*A*
O3—H3···N3^i^	0.83 (2)	1.95 (2)	2.767 (3)	171 (3)
O5—H5···O3^ii^	0.82 (3)	2.02 (3)	2.821 (3)	164 (3)
C7—H7···O1^iii^	0.93	2.59	3.496 (3)	165

Symmetry codes: (i) *x*, *y*+1, *z*; (ii) *x*+1/2, −*y*+5/2, −*z*+1; (iii) *x*+1, *y*, *z*.
